# Reply to Grant, W.B. Comment on “Vicente, F.; Pereira, P.C. Pork Meat Composition and Health: A Review of the Evidence. *Foods* 2024, *13*, 1905”

**DOI:** 10.3390/foods13193044

**Published:** 2024-09-25

**Authors:** Filipa Vicente, Paula C. Pereira

**Affiliations:** Applied Nutrition Research Group (GENA), Nutrition Lab, CIIEM—Egas Moniz School of Health & Science, Caparica, 2829-511 Almada, Portugal; pmpereira@egasmoniz.edu.pt

Our most recent paper, “Pork Meat Composition and Health: A Review of the Evidence”, is, as stated in title, a review [1]. In fact, it is a narrative review written with a clear aim to “analyze and present coherent composition of data on pork meat, establish its possible impact on health outcomes and discuss the presence of this meat option in a sustainable and healthy dietary pattern”. It is not a systematic review, and neither does it imply a causative influence or benefit from pork consumption. It takes pains to clarify that studies in nutrition sciences deserve a careful approach because, as with any other food, pork meat is not an isolated risk or protective dietary factor with respect to disease risk or health outcomes.

It is important to highlight that this review brings up relevant data on one subject that deserves further attention, namely, the differences in pork meat cuts’ composition. In the comment [2], Grant (2024) solely refers to pork meat and its possible pejorative consequences in health without considering the presented differences in fat content and fatty acid profile from different cuts. Considering that, to the best of our knowledge, and according to the most relevant and recent literature, most negative effects in chronic non-communicable diseases are associated with excessive fat, and especially saturated fat consumption [3,4], this should be considered.

Additionally, when addressing the possible influence of pork meat consumption on health outcomes, the authors have only considered clinical trials and randomized clinical trials. Grant (2024) [2] refers to important and relevant studies about healthy dietary patterns and the impact meat consumption, and these are observational studies. However, the sample size and the possible magnitude of the effect should not be ignored; there is a methodologic difference between both types of study and in the type of conclusions that each should address.

Despite the existence of several challenges in conducting RCTs that assess the effects of specific nutritional interventions and diet factors, these clinical trials are still the gold standard for establishing causal relations between exposure to certain nutrients and foods or dietary patterns and specific outcomes [5,6]. Evidence-based dietary guidance is derived from systematic reviews and meta-analyses of this type of study.

In the published review, it was possible to include the very few published studies that could present a clear conclusion on the effects of pork consumption, and several variables should be considered in further studies, namely, the studied outcomes, the sample characteristics, the pork meat portions, and the cuts included. Most epidemiologic data refer to results from food frequency questionnaires, which are adequate for these samples but lack precision when evaluating the consumption of very specific foods. In nutrition research, there are several changes with respect to the design, validation, and use of food frequency questionnaires is always present when one is deciding on the most adequate food intake assessment method [7].

For instance, one considerable limitation in most referenced FFQs is the difficulty in separating meat cuts and types of pork from other meat options. This can increase the item number in the food list, with possible implications for participants’ adherence. Most studies use FFQs, and this tool should be validated for the goal and the food group/food item that is being evaluated [8].

Nevertheless, in the referenced clinical trials, pork meat was included as part of a healthy dietary pattern, though not as the only protein source or meat eaten and can thus be recommended from the point of view of promoting a healthy diet, the recommended approach that is intended to be supported by our research work.

## References

[1] Vicente F., Pereira P.C. (2024). Pork Meat Composition and Health: A Review of the Evidence. Foods.

[2] Grant W.B. (2024). Comment on “Vicente, F.; Pereira, P.C. Pork Meat Composition and Health: A Review of the Evidence. *Foods*
**2024**, *13*, 1905”. Foods.

[3] Kim Y., Je Y., Giovannucci E.L. (2021). Association between Dietary Fat Intake and Mortality from All-Causes, Cardiovascular Disease, and Cancer: A Systematic Review and Meta-Analysis of Prospective Cohort Studies. Clin. Nutr..

[4] Li Z., Lei H., Jiang H., Fan Y., Shi J., Li C., Chen F., Mi B., Ma M., Lin J. (2022). Saturated Fatty Acid Biomarkers and Risk of Cardiometabolic Diseases: A Meta-Analysis of Prospective Studies. Front. Nutr..

[5] Lichtenstein A.H., Petersen K., Barger K., Hansen K.E., Anderson C.A.M., Baer D.J., Lampe J.W., Rasmussen H., Matthan N.R. (2021). Perspective: Design and Conduct of Human Nutrition Randomized Controlled Trials. Adv. Nutr..

[6] Weaver C.M., Hodges J.K. (2021). Designing, Conducting, and Documenting Human Nutrition Plant-Derived Intervention Trials. Front. Nutr..

[7] Willett W.C., Lenart E. (2012). Reproducibility and Validity of Food Frequency Questionnaires. Nutritional Epidemiology 2012.

[8] Cade J., Thompson R., Burley V., Warm D. (2002). Development, Validation and Utilisation of Food-Frequency Questionnaires—A Review. Public Health Nutr..

